# Colo-Vesical Fistula: Relevance of Conventional Radiology in the Modern Era

**DOI:** 10.7759/cureus.36037

**Published:** 2023-03-12

**Authors:** Pooja Ladke, Kajal Mitra, Avinash Dhok, Suruchi Dhawan, Priya Potdukhe

**Affiliations:** 1 Radiodiagnosis, NKP Salve Institute of Medical Sciences and Lata Mangeshkar Hospital, Nagpur, IND

**Keywords:** colo-vesical fistula, barium enema, sigmoid colon, urinary bladder, diverticulosis

## Abstract

Colo-vesical fistula (CVF) is usually encountered in severe inflammatory and malignant conditions. Radiological imaging is crucial to the diagnosis of a colo-vesical fistula and helps gastroenterologists and surgeons select the best treatment option. This disorder is typically identified during follow-up of treatments for diverticulitis or chronic inflammatory bowel disease. The patients present with symptoms of pneumaturia and fecaluria. The most accurate imaging modality for diagnosing CVF is CT with rectal contrast.

In this case report, we present a case of a 58-year-old male patient with complaints of fecaluria and pneumaturia for three months. Ultrasonography and barium enema revealed a clear fistulous tract between the sigmoid colon and the urinary bladder. Subsequently, the patient underwent exploratory laparotomy and the specimen was sent for histopathological evaluation to rule out malignancy. The diagnosis on imaging was consistent with post-operative findings of a CVF. This case report will add to the knowledge of radiologists about the imaging features of CVFs and their identification on imaging.

## Introduction

The term *colo-vesical fistula* (CVF) refers to an abnormal connection between the large bowel and the urinary bladder. Colo-vesical fistulas produce severe morbidity and significantly impact the patient's quality of life. They are most commonly caused by advanced-stage illness or by traumatic or iatrogenic injuries. A CVF diagnosis can be difficult and is sometimes delayed for several months even after recurrent symptoms. Radiological imaging is critical in determining fistulae's location, course, and complexity and detecting an etiological cause. While surgical treatment is the preferred method of management, conservative treatment has been reported to be associated with the same disease-specific mortality as surgery. Conservative management is usually reserved for patients who are unfit for major intervention or have an extensive unresectable neoplastic process. In such cases, medical therapy with catheter drainage of the bladder alone or supra-vesical percutaneous diversion could be beneficial. Surgical management of CVFs involves resecting and re-anastomosing the offending bowel segment and closing the bladder. The treatment may involve single-stage or multistage procedures, depending on the underlying pathology, site of the bowel lesion, and patient’s preoperative status. Bowel resection with primary anastomosis is advocated in the majority of cases. This case report explains the imaging presentations of CVFs and the treatment options available [1].

Diverticulitis, gastrointestinal or genitourinary neoplasms, and inflammatory bowel disease are the most common causes of fistulous connections between the bladder and small or large intestine. It's also attributed to foreign objects, pelvic surgery, and radiation therapy. Although women are at a considerably higher risk of fistulous connection following hysterectomy, mostly males outnumber women [2].

## Case presentation

Patient information

A 58-year-old patient was admitted to the urology department with complaints of burning in micturition, fecaluria, and pneumaturia for three months associated with episodes of abdominal pain. The patient had previous cystitis episodes associated with dysuria, fecaluria, and pneumaturia. The patient was advised antibiotics by a general practitioner but did not have any relief of symptoms.

On examination, the patient was vitally stable. He was given ceftriaxone and metronidazole to relieve dysuria and stomach discomfort, but the fecaluria and pneumaturia persisted.

Diagnostic assessment

Laboratory tests revealed blood values of white blood cell count of 11.360; hemoglobin 12.4; urea: 74 mg/dL (raised); creatinine: 2.5 mg/dL (raised). Abdominal contrast-enhanced computed tomography (CECT) was contraindicated in our patient due to raised serum creatinine. The patient was referred for USG followed by a barium enema for further evaluation. 

Clinical findings

**Figure 1:**
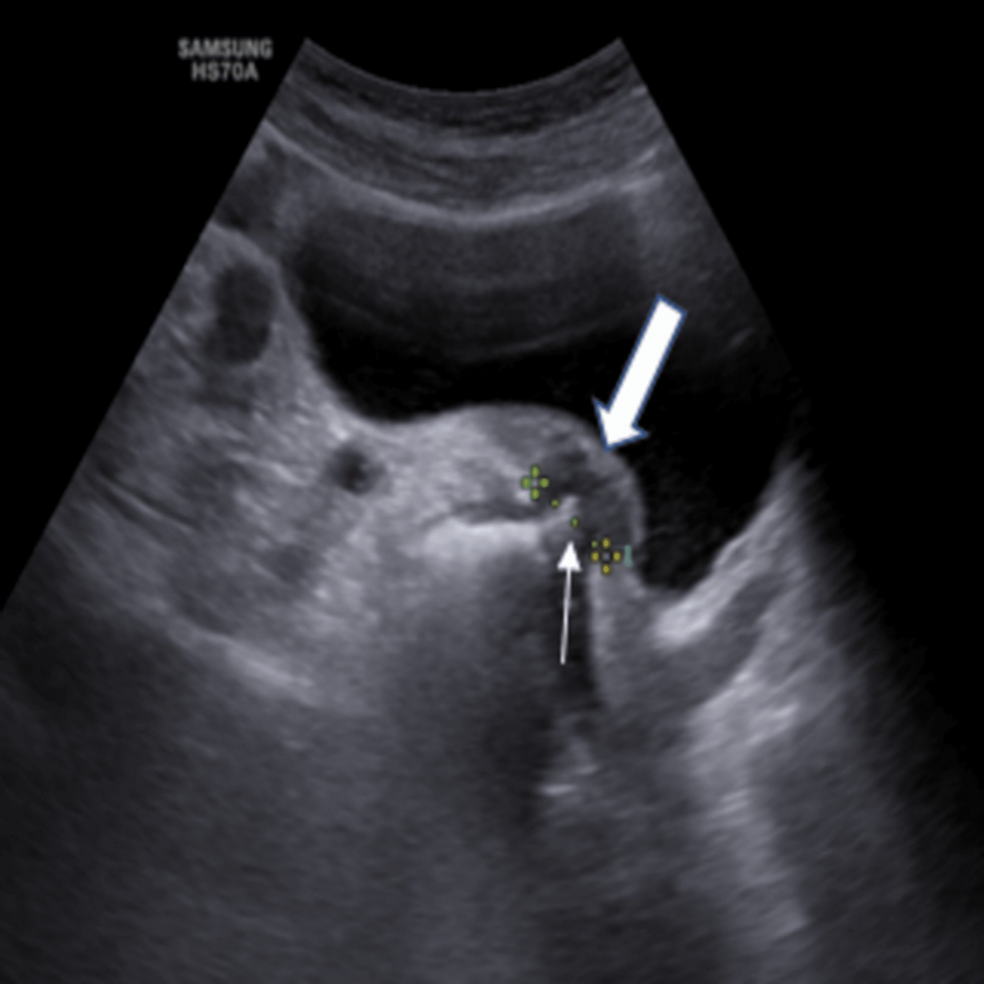
Ultrasonography axial section through the urinary bladder shows a fistulous connection between the sigmoid colon and urinary bladder (block arrow) through a defect (line arrow)

On scanning through the urinary bladder on USG (Figure 1), the passage of echogenic material into the urinary bladder was seen through an approximate 1.5 cm defect between the sigmoid colon and the urinary bladder. This was distinguishable from the episodic, bilateral jets of urine from the ureteric orifices. These findings confirmed the diagnosis as well as the location of the fistulous connection between the sigmoid colon and urinary bladder. As an incidental finding, the patient also had a 2 cm calculus in the mid-ureter on the left side. 

**Figure 2:**
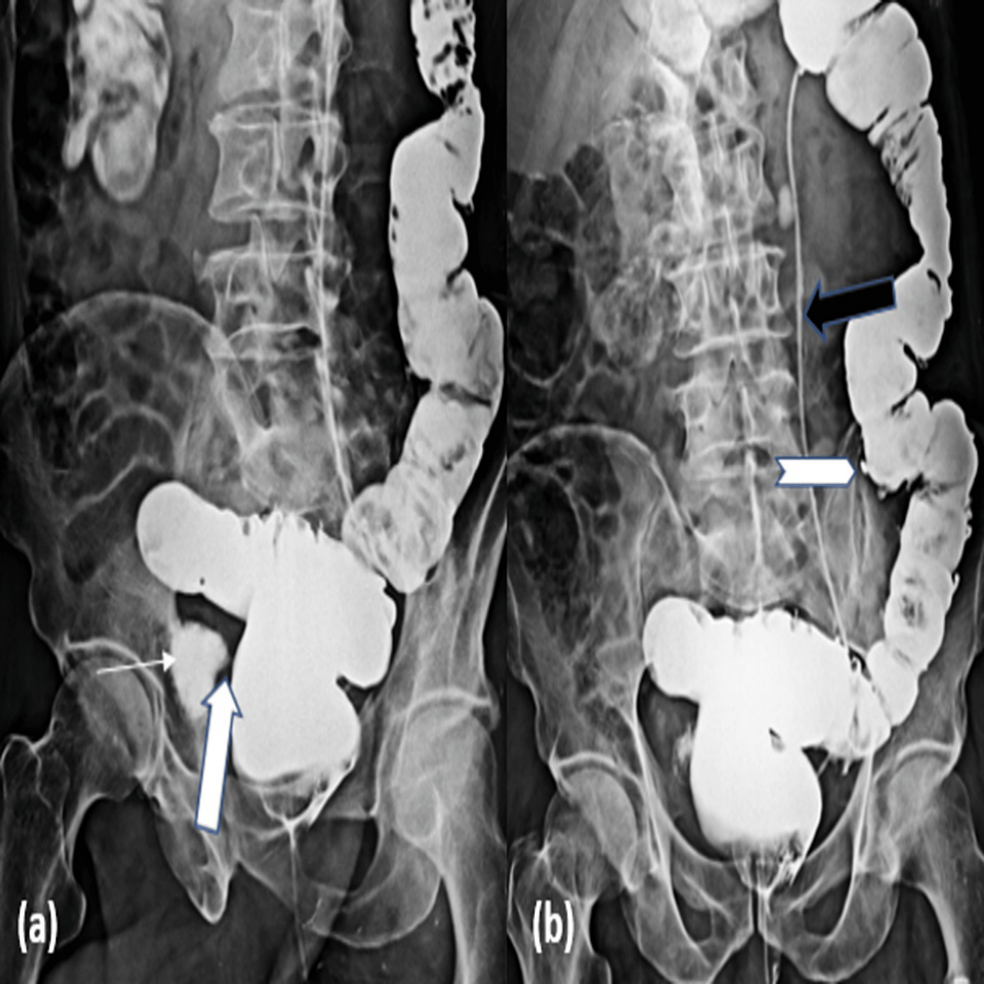
(a) Barium enema showing leakage of contrast into the urinary bladder (line arrow) through a fistulous connection between the sigmoid colon and urinary bladder (block arrow);  (b) Multiple diverticula (chevron arrow) are seen along the descending and sigmoid colon. The double J (DJ) stent is also noted on the left side (black arrow).

A barium enema (Figure 2) was performed to demonstrate the passage of the barium from the sigmoid colon into the urinary bladder. In addition, barium enema revealed multiple diverticula along the lumen of descending and sigmoid colon which could not be appreciated on USG of the abdomen.

Diagnosis & therapeutic intervention

**Figure 3:**
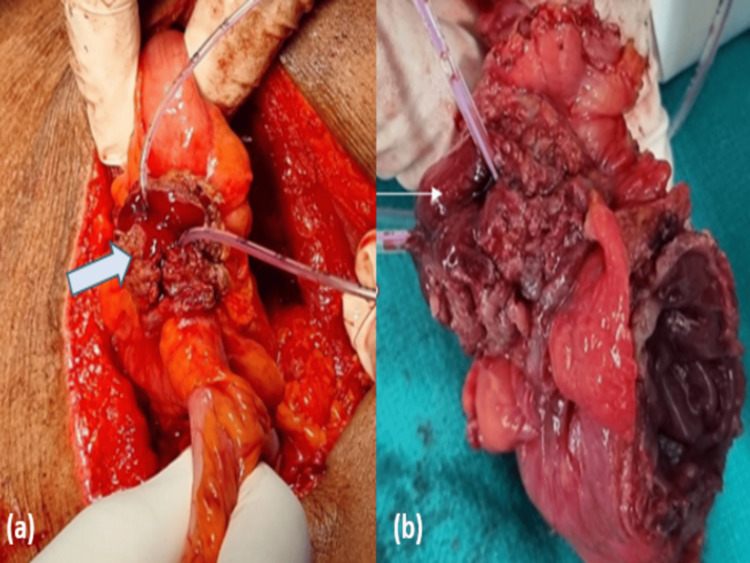
(a) Intraoperative image showing adherences between the sigmoid colon and the urinary bladder demonstrated by the through and through passage of the Ryles tube (white arrow);  (b) Postoperative image showing the necrotic tissue in the sigmoid colon (line arrow).

The diagnosis of entero-vesical fistula due to diverticulosis was considered in view of imaging findings. The patient underwent exploratory laparotomy, which revealed significant adherences between the sigmoid colon and the urinary bladder (Figure 3 a). The sigmoid colon was carefully separated from the posterior wall of the urinary bladder. Along the connection, necrotic tissue was also evident along with multiple diverticula (Figure 3 b). A recto-sigmoidectomy of approximately 15 cm was done along with a proximal segment colostomy. The dissected tissue was sent for histopathological examination which revealed a non-specific chronic inflammatory process consistent with diverticulitis. Malignant cells were not demonstrated.

## Discussion

Entero-vesical fistulas (EVFs) most commonly occur in the setting of inflammatory bowel disease. With diverticulosis, there is a 1% to 4% relative risk of having an EVF. There are two different types of EVF: (1) ileo-vesical fistula, and (2) colo-vesical fistula. The commonest cause of CVF is diverticulitis. The underlying cause is frequently a ruptured diverticulum or an abscess that has invaded the urinary bladder's peri diverticular space. Phlegmon and abscesses are risk factors for the formation of an impending fistula [1]. 

The majority of the ileo-vesical fistula is caused by Crohn's disease, which also explains why individuals with ileo-vesical fistula typically present at a considerably younger age. Neoplasm or trauma are generally the causes of rectovesical fistula [2]. 

While pneumaturia is suggestive and fecaluria is pathognomonic, patients frequently arrive with non-specific symptoms that cause a delay in diagnosis. To make the diagnosis in these situations, additional early investigations are required. A CVF can also be diagnosed by other laparoscopic procedures such as cystoscopy, colonoscopy, cystography, and cross-sectional imaging [3].

Pneumaturia, fecaluria, fever, and recurrent cystitis are among the clinical signs and symptoms of CVF. Until about a decade ago, the primary radiological tests were barium enema and cystography. Cross-sectional imaging has now surpassed them. Both CT and MRI have the advantage of establishing the underlying etiology, fistula form, and anatomical location of the tract [4]. 

Both conservative and surgical therapy are available for CVF treatment. This abnormal communication has been treated surgically using both open and laparoscopic procedures. Gastrointestinal tract anastomosis and bladder reconstruction are aimed at surgically removing the adhesions between the colon and bladder. The most severe complication of the CVF fistula is sepsis, which can be fatal in patients who are left untreated [5]. The presence of colonic diverticula, the air in the bladder, and the thickening of the bladder wall close to the loop of the thickened colon are the three signs that raise suspicion of a CVF [6].

A potentially useful way of visualizing the CVF is transabdominal sonography paired with abdominal compression. Additional investigation is needed to demonstrate its diagnostic accuracy in comparison to conventional procedures [7].

When managing CVF surgically, less invasive methods are the preferred options. Patients with benign and non-radiation-induced CVF are less likely to experience their fistula recurring after surgery. Its occurrence should raise the suspicion of underlying cancer, especially if the surgery was well-performed [8].

## Conclusions

The case report of a CVF describes an unusual complication of diverticulosis of the colon in which the role of conventional radiology is still important in the modern era for confirmatory diagnosis. A fistulous connection between the sigmoid colon and urinary bladder was identified on barium enema as well as USG.

The patient underwent surgical resection of the sigmoid colon which was containing the fistula. Histologic examination confirmed an underlying inflammatory process. No evidence of recurrence was noted on post-operative follow-up. The importance of conventional radiological imaging in the diagnosis of CVF and its pathogenesis is facilitated by this case report.
